# Technical Readiness and Stereotypes in Hospital Nursing—A Question of Gender and Age?

**DOI:** 10.3390/nursrep13010013

**Published:** 2023-01-22

**Authors:** Lisa Korte, Sabine Bohnet-Joschko

**Affiliations:** Chair of Healthcare Management and Innovation, Faculty of Management, Economics and Society, Witten/Herdecke University, 58455 Witten, Germany

**Keywords:** nursing staff, gender, age, innovation, technical readiness, survey

## Abstract

(1) Background: The nursing profession is associated with various stereotypes. These social images or prejudices against specific groups can inhibit the personal growth of individuals, e.g., sociodemographic characteristics influence the social image of nurses. Based on the forward-looking topic digitization, we examined and discussed the influences of sociodemographic characteristics and motives of hospital nurses on technical readiness to gain insights into the digitization process in hospital nursing. (2) Methods: As part of an online survey on technical readiness among German hospital nurses, we particularly examined sociodemographic influences on technical readiness and the relationship between sociodemographic characteristics and professional motives. Furthermore, we included a qualitative analysis of optional comment fields. (3) Results: The analysis included 295 responses. Age and gender had a significant influence on technical readiness. Furthermore, the importance of motives differed between gender and age. The analysis of the comments produced three categories specifying our results: beneficial experiences, obstructive experiences and further conditions. (4) Conclusions: In general, the nurses showed high technical readiness. In order to gain high motivation for digitization and promote personal growth, special targeting and cooperation between gender and age groups can be beneficial. However, there are more sites at system level, such as funding, cooperation and consistence.

## 1. Introduction

There exist various stereotypes in the nursing profession [1,2]. Stereotypes are characteristics and behaviors describing a specific group. They are closely associated with gender and further sociodemographic factors [3]. The social images can influence the work of nurses and inhibit their personal growth [1,4]. In the past, nursing became a profession especially associated with gender convictions. Studies pointed out a social image describing the nursing profession as a female and caring activity [1,3,5]. The stereotypical prejudices that nurses just perform caring tasks are a barrier for any expansion of their activities and responsibilities [3,6], especially important in the context of digitization and digital competencies. The same applies to the altruistic, empathic and traditional role model of nurses [5]. Furthermore, there are also judgments about less competence among older nurses [7,8]. Age stereotypes mean general assumptions regarding characteristics and skills in certain age groups. In the nursing profession, the age range is very large. For example, a study showed that old nurses have the role of being honest and trustworthy but are also less open to changes and learning. Especially in context of current changes such as digital transformation processes, these prejudices can restrict technical readiness of older nurses and be an additional stressor [7], even though there is an increasingly potential for extension of nurses, and their responsibilities and competences [2,3]. Therefore, critical handling of nursing stereotypes and awareness raising is crucial [1,3]. By strengthening individual nurses and through cooperation between different sociodemographic nursing groups, nurses can become more confident and engaged in the process of digitization [1]. Technical readiness of nurses is an increasingly significant element. Digital transformation offers great potential for addressing the shortage of skilled nurses and the ongoing COVID−19 pandemic, particularly in large hospitals [9,10]. Innovative technologies can provide timely care for patients while easing the burden on nurses [11,12,13]. However, their implementation and use first requires expense and new competencies in different nursing groups who may be insecure due to sociodemographic prejudices [14,15,16]. A successful implementation and use of new technologies requires diversity management and a target-oriented understanding of nurses’ technical readiness in order to design differentiated implementation processes that address individual needs of nurses. Simultaneously cooperative approaches can support reducing gaps in digital competence and confidence [7,11,17].

### Aims and Hypotheses

The aim of this study was to analyze influences on the technical readiness of German hospital nurses. In particular, we investigated stereotypical assumptions influenced by sociodemographic characteristics and other potential influencing factors on technical readiness of hospital nurses. The results and the understanding of effects on the nursing staff’s technological readiness can increase a critical handling with stereotypes as well as awareness. They can contribute to the development of a need- and target group-oriented approach when introducing and using new technologies in hospital nursing. Therefore, we also examined sociodemographic differences in the importance of different professional motives for the nurses. Based on a systematized literature review and intensive consultation with nursing experts (nurses, nursing leaders, researchers), we developed the following hypotheses:

**H1:**  *Gender has an influence on the technical readiness of hospital nurses.*

**H2:**  *Age has an influence on the technical readiness of hospital nurses.*

**H3:**  *Educational background has an influence on the technical readiness of hospital nurses.*

**H4:**  *Sociodemographic characteristics cause differences in the significance of professional motives for hospital nurses.*

## 2. Materials and Methods

### 2.1. Study and Questionnaire Design

We conducted a study on technical readiness in hospital nursing. In addition to vignettes regarding the introduction of two specific digital innovations in hospital nursing, which are not subject here, the study included survey instruments on technical readiness and job satisfaction as well as a query on sociodemographic, other personal and work-related characteristics. We conducted the web-based online survey via the academic online survey tool LimeSurvey. The target group consisted of German hospital nurses. Regarding technical readiness and job satisfaction, we chose the short scales on general willingness to use technology and job satisfaction from the compilation of social science items (“ZIS”) of “GESIS–Leibniz Institute for the Social Sciences e.V.” [18,19]. Other international studies also used these scales before [20,21,22,23]. We added questions regarding possible influences based on intensive consultation of nursing experts (nurses, nursing leaders and researchers). The questions about the person, for example, covered the use of digital technologies, such as a tablet, in private and professional everyday life. The work-related characteristics included questions about the professional background, the workplace, and the importance of specific work motives. For the motives, we selected basic intrinsic and extrinsic motivation (IM and EM) [24,25,26] and patient and efficiency orientation (PO and EO) as significant values in hospital nursing [27,28,29]. We developed and tested phrases that displayed IM as motivation for an interesting and enjoyable task and EM as motivation stimulated by external reward [24,25,26]. The terms for IM and EM were “interesting activities” and “expansion of professional competencies and opportunities” [24,25,26,30,31,32], for PO and EO “more time for individual patients” and “completing tasks more quickly” [27,28,29,33,34,35]. Except for an open comment option at the end of the questionnaire, we specified all answer options, mainly by rating on a six-point Likert scale. The questions of the survey are provided in the Supplementary Materials.

### 2.2. Recruitment

We distributed the online survey between 24 November 2021 and 20 January 2022. We spread the link in personal social networks, on social platforms, and via email distribution lists for related staff and experts with further connections. Several hospitals and networks of professionals also distributed the survey. In this step, we contacted nursing directors via the German hospital directory. We referred to very large institutions because the process of digitization is very advanced here and so we could reach as many nursing professionals as possible.

### 2.3. Data Processing and Statistical Analysis

We cleaned the raw data set in Microsoft Excel by removing incomplete and erroneous questionnaires. Since all crucial questions referred to non-sensitive and mandatory data, there were no missing values. Other questions had answer options such as “not specified”. We excluded data sets for methodological reasons, when respondents chose these fields within the main variables to make regression analysis possible and easier. We applied this to the education status, because it affected only four persons. We coded data in text format into numeric indicator variables and created dummy variables for nominal variables in order to use them in the regression analysis.

We used two short scales on general willingness to use technology and job satisfaction from the compilation of social science items (“ZIS”) of “GESIS–Leibniz Institute for Social Sciences e.V.” [18,19]. The individual items were coded in the same directions in order to define a low willingness to use technology or low job satisfaction with low numbers. For both scales, we calculated new variables to indicate the average values. We categorized variables with many expressions, such as age, in groups to facilitate a presentation of descriptive statistics. We conducted the analyses with the origin metric variable of age.

Regarding influences on the technical readiness of hospital nurses, we used correlation analyses and mean comparisons as one first step to determine whether differences existed at all. Regarding H1 to H3, the sociodemographic variables age, gender and educational background were crucial. We used a regression model to examine all surveyed potential influence factors on technical readiness. Regarding H4, we applied correlation analyses and mean comparisons between sociodemographic factors and motives in order to exploit possible correlations for a target-group-specific approach. The independent variables included in the regression model for technical readiness were as follows:

− Age;
− Gender;
− Educational background;
− Frequency of professional tablet use;
− Frequency of private tablet use;
− Degree of digitization–hospital;
− Degree of digitization–department;
− Ownership of the hospital;
− Number of beds in the hospital;
− Specialization/further education;
− Personnel responsibility;
− Scope of employment;
− Work experience in nursing;
− Work experience at current workplace;
− Age of leader;
− Gender of leader;
− Job satisfaction;
− Importance of IM in everyday work;
− Importance of EM in everyday work;
− Importance of PO in everyday work;
− Importance of EO in everyday work.

In order to specify our results, we classified the open comments at the end of the query.

We disregarded the comments that did not include content regarding digitization in hospital nursing, e.g., “good luck” or methodical aspects.

## 3. Results

### 3.1. Sample—Respondent Characteristics

We removed 232 incomplete questionnaires that people only clicked one time or that had no useful information and 17 data sets that did not meet the inclusion criteria of being a nurse and working in a hospital. In the end, 295 data sets remained. Around three quarters of the sample were female (227). The average age was 37 years. The youngest person was 18, the oldest 63 years old. The largest age group was age 20 to 29 years (32%). Most participants had a high school diploma (“Abitur”) (38%). The respondents’ technical readiness was generally high. The nurses selected the upper values 4 to 6 more frequently (91%). Table 1 presents sociodemographic characteristic as well as the values for technical readiness.

#### 3.1.1. Sample—Profession and Workplace

Overall, 80% of the participants were part of the general professional health care and nursing group. Almost a quarter of them had an academic nursing degree (23%). The sample was distributed across various specialties. A high proportion (21%) of respondents worked in intensive care medicine. The average scope of employment was 84%, with about 55% working full-time. The work experience of the respondents at the current job was an average number of nine years, their total work experience nearly 18 years. Almost 22% had personnel responsibility and more than half of the participants worked in large hospitals with at least 800 beds (59%). More than two-thirds of respondents worked at hospitals under public ownership (71.5%). Table 2 summarizes key data regarding the workplace.

#### 3.1.2. Sample—Professional Motives 

All characteristics (IM/EM/PO/EO) were important for most respondents in their everyday working lives. The nurses selected the upper values 4 to 6 more frequently than 1 to 3. We found the highest percentage of the three upper values for PO (99%) and IM (96%). For EM, it was 90% and for EO 78%. Median and mode were also higher for IM and PO (6), than for EM and EO (5).

### 3.2. Results: Technical Readiness in Hospital Nursing

Regarding H1–H3, we first conducted correlation analyses between the sociodemographic variables and technical readiness separately from the regression model. For age and gender, correlation analyses and mean comparisons indicated significant relations. The application of the Mann–Whitney U-test (Z = −3.652, *p* ≤ 0.001) and a bivariate correlation analysis between technical readiness and gender using the Chi² test (*p* = 0.05) showed that male respondents had a higher technical readiness than female. A group comparison of mode and median likewise revealed gender differences. While both scores were 5 for male respondents, they were 4 for female. A bivariate correlation analysis between age and technology readiness using Spearman rho further demonstrated a significant relation (ρ = −0.136, *p* = 0.010). For educational background, the same analyses did not demonstrate a significant relation with respondents’ technical readiness.

We performed a more precise operationalization of these relations in consideration of other influencing factors using multiple regression analysis. The model explained with around 12% a rather small portion of the participants´ technical readiness (corrected R² = 0.121). Age (B = −0.016, *p* = 0.013) and gender (B = 0.374, *p* = <0.001) also showed significant values in the regression model. Furthermore, the frequency of private tablet use (B = 0.076, *p* = 0.007) and the degree of digitization in the department (B = −0.098, *p* = 0.029) showed a significant influence on the technical readiness of the respondents. The model suggested a fifth variable with a significant influence, the hospital ownership. Using non-profit/denominational ownership as the reference category, a difference from the dummy variable of private ownership was demonstrated (B = −0.481, *p* = 0.041). However, due to a very unequal distribution, we did not consider the influence of ownership in further progress.

Overall, we could confirm **H1** and **H2**: the analyses showed that gender and age had an influence on the nurses´ technical readiness. It was higher among male and younger respondents. We could reject **H3**: the educational background did not have an influence on the participants’ technical readiness. Table 3 summarizes key data regarding the significant influences for technical readiness.

### 3.3. Results: Significant Motives in Hospital Nursing

We found significant differences by analyzing the correlations between the motives (IM/EM/PO/EO) and sociodemographic factors. A bivariate correlation analysis between age and IM using Spearman rho indicated a significant relation (ρ = −0.165, *p* = 0.002). Consequently, IM appeared to be more important to the older nurses than to the younger participants. For gender and PO, the Mann–Whitney U-test (Z = −3.438, *p* ≤ 0.001) and a bivariate correlation analysis using a Chi² test (*p* = 0.006) demonstrated a significant relation. Post hoc analyses showed that more female respondents (80%) than male ones (50%) selected the highest category “very important” when evaluating their importance of PO. Women, thus, seemed to consider PO more important than men did. Finally, we could confirm **H4**: Sociodemographic characteristics of the respondents indicated differences in the importance of professional motives; older participants seemed to consider IM particularly important as well as women PO.

### 3.4. Results: Supplementary Qualitative Comment Analysis

In order to complement our analysis, we sifted the open comment fields of the survey and developed three categories that describe the attitude of the respondents regarding digitization in hospital nursing and further circumstances. The first category describes *beneficial experiences* the nurses already made. These seemed to enhance their motivation in context of using digital innovations at work as well as their confidence towards the digital developments and the advantages. The statements in this group connected digitization in hospital nursing with saving of time, facilitation in daily work and flexibility. In addition, the nurses hoped for better networking, cooperation and exchange—especially between different professions, teams and departments—through digital elements and the related steady data access and transparency in the working day. Some participants also associated digital possibilities with a higher patient safety and an expansion of the professional nursing image—due to changes in form of learning, developing new competencies and through better knowledge transfer. An example for a comment in this category is “digitization in care can be advanced much more quickly, as I believe it simplifies and shortens work steps and can also help caregivers have a better overview of the people in need of care.”

The second category contents the opposite of the first one. In the group of *obstructive experiences*, we collected statements that described the defense of some respondents. This means an attitude that is skeptical and pessimistic towards digital innovations in hospital nursing. Reasons were previous encounters with digital systems and devices that do not function properly, were defective or incompatible. Furthermore, the negative attitude raised from experienced time-consuming training with digital innovations. Disadvantages mentioned in the statements of this category were also financing insecurity and difficulty as well as a lack of participation. An example comment for this category is “digitization to date has done nothing to simplify or improve the work of nurses. It takes up significantly more time. The management attaches great importance to the completeness of the documentation...thus the few specialists spend even less time with the patient.”

The third, and final, category of our qualitative analysis is like a realization of the other two groups. It describes *conditions, expectations and wishes* of the nurses, particularly with regard to digitization in hospital nursing, but also in a larger context. Furthermore, this category considers both, an individual and system level. Contents mentioned by the participants are the need for training especially for older nurses who are insecure regarding new technologies: “…, but the older ones must not be forgotten”. This includes constant support and availability of responsible firms and people when there are questions or problems. There existed the assumption that older and experienced nurses had a defensive attitude towards digital innovations. That is why, according to the participants, the systems and devices should be user-friendly and technical requirements and infrastructures are crucial. Another necessary condition is the cooperation of different involved stakeholders, containing uniform rules and standards as well as participation. Overall, there was the wish to follow a focus on the patients and their care. Regarding this, a comprehensive strategy is required, as well as adaption, practical training and learning. A sense of responsibility for these changes was also an element of this category. However, the assumption that digitization is not a solution for systematic and bigger problems in the health care system arose. This comment presents part of the third category: “I would like to see better linking of the individual documentation systems so that all professions only document in one system.” In the following, we will discuss our results and the described comments of the participants. 

## 4. Discussion

We analyzed 295 data sets from German hospital nurses. It is important to emphasize that the technical readiness of the respondents was generally high. Around 90% of the surveyed nurses chose the three upper answer options of “rather high”, “high” and “very high” technical readiness. However, we identified significant influencing factors on the nurses´ technical readiness. These consisted of age and gender, as well as digital experiences. The differences can serve to use appropriate target group-oriented communication and cooperation in order to increase engagement and motivation when implementing digital innovations. This can further address challenges such as consequences of the COVID−19-pandemic and general demographic developments [10,17,36].

### 4.1. Promising Conditions for Digital Hospital Nursing

In general, the technical readiness of the nursing respondents was high, which is a promising result for future developments of digitization in hospital nursing and an expansion of the nursing profession and responsibilities. The analysis of sociodemographic factors revealed small differences: there was a higher technical readiness among the male and younger nurses. These findings are consistent with the literature and existing stereotypes about less digital literacy and technical readiness among older and female nurses [14,15]. It is, therefore, essential to provide confidence and competence in dealing with digital innovations–based on specific needs and through gender- and age-specific interventions as well as group cooperation [14]. It is important to reduce prejudices in order to support personal growth in the nursing profession and emphasize individual strengthens. Cooperation between the different gender and age groups and a comprehensive diversity management are crucial [6,7]. Our findings set out that there are promising conditions. The surveyed nurses were generally motivated and already worked in their profession for almost 20 years and at their current workplace for almost 10 years on average. Furthermore, around 20% had personnel responsibility and around 30% had an academic degree. This underlines the extension of the nursing profession and can be good starting point for further development of responsibilities and new tasks fields.

Preexisting access to and regular use of new technologies is conducive to technical readiness [15]. Respondents with more frequent private tablet use seemed to be more confident in using such technologies and, therefore, have less inhibition than participants with little technical contact. Professional training that promotes digital skills can be beneficial and can create a general acceptance of innovation and change. This is especially promising since the respondents seemed to have a high technical readiness, contrary to general assumptions [16] and supported, in particular, by the category *beneficial experiences* of the qualitative analysis.

The negative correlation of technical readiness with the degree of digitization in the specialty department could result from the fact that the participants made bad experiences with the systems and products previously used. Maybe the technologies did not work or were not user-friendly. The comment category of the *obstructive experiences* with digitization confirmed this, as well as findings from the literature [37,38]. Otherwise, the respondents saw potential in digitization. They might be only less optimistic due to opposite experiences. Increasing practical experience and participation in the context of projects, personnel development measures and workshops can be beneficial and are mentioned in one category of the qualitative analyses; the one describing *conditions, expectations and wishes*. In this way, acceptance barriers might decrease and responsible professionals can pursue a solution oriented error culture [37]. This can succeed if they simultaneously address staff individually and support cooperation of different sociodemographic groups. However, we have to consider that the multiple influences on the nurses´ technical readiness make a complete representation of this variable almost impossible. That is why the regression model explained only 12% of technology readiness. In order to support the nurses´ confidence and competence regarding digitization in hospital nursing in context of pandemic burdens and nursing shortage [36,39,40,41], further actions at the organizational and structural macro level are crucial. Finally, it is worth mentioning that only women and men were participants of our survey and conclusions refer to these two genders. In a broader sense, it is important to advance gender-sensitive research that includes diverse identities. This is the only way to deal critically and reflectively with general stereotypes in nursing and to support further development of the profession [1,2,3].

### 4.2. The Opportunities of Different Motivational Paths

The role of different job motives varied among the respondents and differed between age and gender. Older respondents seemed to care more about IM. This is, in a sense, consistent with the altruistic, trustworthy and honest social image of older nurses [7]. Younger people strive for fulfillment and sense, but still have a desire for recognition, are in search of identity, and grow up with high social pressure [42]. For example, financial incentives, career options and autonomy are also important to them [7,43]. Thus, literature findings show a higher sense of stress among younger professionals: They perceive job-related demands and uncertainties as more stressful than older comparison groups and are more susceptible to mental health problems [44]. IM particularly promoted resilience. Therefore, it is crucial to increase its role in younger nurses so that they can meet the demands of everyday professional life and remain in the profession. For the female respondents, PO was more important. This is consistent with stereotypical and conventional assumptions that women place high value on relationship work, reflected in the high percentage of women in nursing professions [3,25,45]. The findings regarding the motives are in line with the nursing stereotypes and roles [1,3,7]. This demonstrates the urgency of cooperation, especially between different age and gender groups, in order to reach similar level of confidence and competence and to show each other the importance of different motives in hospital nursing. At the same time, it is important to emphasize individual strengthens and promote diversity management [7]–because the respondents showed up the differences between the age groups regarding attitudes towards digitization in the category of the *conditions*.

Finally, hospital nursing leaders can address and motivate their staff at different levels in order to support personal growth but should also focus on collaboration [25]. Accordingly, stereotypical assumptions can be a starting point, because they are deeply anchored in society [3]. In this context, communication should be target group-specific and consider professional guiding principles. This is particularly helpful in the context of continuous change and adaptation processes [46]. The complexity of the effect of motivation and the several possible influences make general statements difficult. Intervention in context of human resource development such as communication training can improve the understanding of the nurses´ motivation and enable leaders to adapt their behavior.

### 4.3. Limitations

Various personal and work-related factors were part of the survey and analysis. Nevertheless, important potential influencing factors may have been missing in order to explain technical readiness of hospital nurses better. We recruited respondents primarily through digital media, which might have led to self-selectivity in participation and might have led to a respondent group of nurses with high technical skills compared to the basic nurse population. Many participants were young and educated and, therefore, covered only part of the hospital nursing staff population. Similarly, more than 50% of the participants worked in large hospitals with 800 or more beds. Another limitation of the study is that it was a self-report survey. Therefore, we could not distinguish between the respondents´ assessment of their own technical readiness and skills and their real commitment. In addition, we could not make sure that the participants indeed were nurses working in a hospital. Finally, a limitation of the results can be the query of motives (IM/EM/PO/EO). Participants could have interpreted the wording differently, despite intensive preparatory steps. For example, not all respondents might have understood the expression “interesting activities” as an intrinsic motive, just like the expressions for the other three motives, and this was only one expression for intrinsic motivation. We had to present a short and easy understandable wording, although the motives are comprehensive constructs with much more content and descriptions.

## 5. Conclusions

Overall, sociodemographic differences and related stereotypes certainly characterize the nursing profession. Our study showed, particularly, the influence of age and gender on the technical readiness of hospital nurses. However, the sociodemographic characteristics are small, whereas the nurses´ technical readiness was generally high, which is a promising condition for digital developments in hospital nursing. Existing sociodemographic differences should not lead to prejudices and point out different competencies or strengthens and weaknesses. Responsible people in hospital nursing can use them in order to develop different interventions and support individual strengthens. Through target group-oriented actions of personnel development, it is possible to respond to the nurses´ needs in an age- and gender-sensitive way and increase group cooperation [47]. For example, external training in the usage of digital innovations can increase the self-security of nurses with little technical experience. Practical experiences in the professional life, such as in context of projects, can mediate specific advantages of the use of digital products to nurses that already feel competent regarding digitization but need to see the benefits. Finally, responsible people in hospital nursing can address different sociodemographic groups at different points; for instance, old people who are already intrinsically motivated need training that is more external, whereas young people have the need of finding extrinsic incentives such as opportunities for advancements. Likewise, the groups can mediate the importance of the various motives to each other. Since the technical readiness was generally high among the participants, continuous motivation is promising. To meet the challenges of the nursing workforce, it may be just as important to promote a general culture of readiness for change beyond expanding competencies. Positive and more experiences with digital innovations can help to mediate skills and security for change processes. Regarding this, the increased implementation of practical projects is promising and can also expand and strengthen the professional nursing image.

## Figures and Tables

**Table 1:** Baseline characteristics of participants.

Variable	N (in Total = 299)
**Gender ** Male Female **Age** (**$\overset{-}{\text{X}}$ = 37 years**) Under 20 years 20–29 years 30–39 years 40–49 years 50–59 years Over 60 years **Highest level of education ** Lower secondary school diploma Secondary school diploma General qualification for university entrance (Technical) College degree **General technical readiness** Very low (1) Low (2) Rather low (3) Rather high (4) High (5) Very high (6)	68 (23%) 227 (77%) 4 (1%) 94 (32%) 77 (26%) 58 (20%) 52 (18%) 10 (3%) 2 (0.5%) 82 (28%) 112 (38%) 99 (33.5%) 0 (0%) 2 (1%) 24 (8%) 122 (41%) 118 (40%) 29 (10%)

**Table 2:** Baseline characteristics of the respondents’ workplaces.

Variable	N (in Total = 299)
**Ownership ** Public Non-profit/denominational Private Do not know exactly **Number of beds ** Less than 100 Less than 200 Less than 300 Less than 400 Less than 500 Less than 600 Less than 700 Less than 800 800 or more Do not know exactly	211 (71.5%) 53 (18%) 14 (4.5%) 17 (6%) 6 (2%) 20 (7%) 6 (2%) 8 (3%) 22 (7.5%) 19 (6%) 11 (4%) 6 (2%) 175 (59%) 22 (7.5%)

**Table 3:** Significant factors in the regression analysis for technical readiness.

Factors	B (*p*)
Gender Age Frequency of private tablet use Degree of digitization in the department	0.374 (<0.001) −0.016 (0.013) 0.076 (0.007) −0.098 (0.029)

## Data Availability

The datasets used and/or analyzed in the context of this study are available from the corresponding author on reasonable request.

## Supplementary Materials

The following supporting information can be downloaded at: https://www.mdpi.com/article/10.3390/nursrep13010013/s1.
