# Supplementary material for: Technical Readiness and Stereotypes in Hospital Nursing—A Question of Gender and Age?
Source: Nurs Rep. 2023 Jan 22;13(1):116–27. doi: 10.3390/nursrep13010013 (PMC9944579; doi:10.3390/nursrep13010013)
Supplement: Supplementary file 1 [file nursrep-13-00013-s001.zip › informed consent.pdf]

Data protection information according to Art. 13 DSGVO:

This online survey of the UW/H is conducted as research work at the Chair of Healthcare Management and Innovation, Faculty of Management, Economics and Society, Witten/Herdecke University. The responsibility for the content lies with Prof. Sabine Bohnet-Joschko.

Specific contact person is: Lisa Korte; lisa.korte@uni-wh.de

The processing of the survey data is solely for the purpose of scientific research. The research question is: What motives and values influence digitization in hospital nursing?

Only the information you provide directly to us via the online survey fields will be included in the research dataset. Connection data that is additionally transmitted in the background by your terminal device for technical reasons is stored separately from the research data. They are only processed for a short period of time (e.g. for system maintenance and troubleshooting purposes) and only by the system administrator. This data is generally excluded from processing for research purposes.

Survey data will be retained only as long as necessary to fulfill the specific research purpose. This is expected to last until 12/2023. After that, the data will be deleted or anonymized in such a way that a reference to your person can no longer be established. Any further processing of personal data or transfer to third parties outside the UW/H is not intended.

The legal basis for the processing of the research data is your voluntary consent according to Art. 6 (1) a DSGVO, which you actively express by clicking the button below and calling up the first page of the online survey. UW/H processes the additional background data in accordance with Art. 6 (1) f DSGVO in its own legitimate interest, but exclusively for the purpose of maintaining its web systems.

You can revoke your consent to the processing of your survey data at any time with effect for the future without any adverse consequences for you. To do so, please contact the above-mentioned contact person.

Please note that a deletion request can only be fulfilled for data that can be clearly assigned to your person.

Further general information on UW/H data protection can be found at: https://www.uni-wh.de/datenschutz/

There, in addition information on data subject rights, you will also find detailed information on the connection data that is transmitted to the UW/H in the background together with the survey data.

You can reach the UW/H data protection officer at datenschutz@uni-wh.de
