# Supplementary material for: Technical Readiness and Stereotypes in Hospital Nursing—A Question of Gender and Age?
Source: Nurs Rep. 2023 Jan 22;13(1):116–27. doi: 10.3390/nursrep13010013 (PMC9944579; doi:10.3390/nursrep13010013)
Supplement: Supplementary file 1 [file nursrep-13-00013-s001.zip › Survey questions.pdf]

Digitization in hospital care

After an introduction text explaining the vignettes, the questionnaire contains additional questions that the respondents could check as follows:

Questions about the person

- **Gender:**

  Male ☐
  Female ☐
  Diverse ☐

- **Age (in years):**

  ____

- **Highest level of education:**

  Lower secondary school diploma ☐
  Secondary school diploma ☐
  General qualification for university entrance ☐
  (Technical) College degree ☐

How often do you use tablets/iPads or similar devices?

	1	2	3	4	5	6
Professional						
Private						

(1=never; 6=daily)

Technical readiness – How would you rate the following statements?

	1	2	3	4	5	6
I am very curious about new technical developments.						
For me, dealing with technical innovations is usually too much of a challenge.						
I find dealing with new technology difficult - I just cannot do it most of the time.						
It is up to me whether I succeed in using new technical developments - this has little to do with chance or luck.						
I am always interested in using the latest technical equipment.						
When dealing with modern technology, I am often afraid of failing.						
If I have difficulties in dealing with technology, it ultimately depends on me alone to solve them.						
If I had the opportunity, I would use tech products much more often than I currently do.						
I'm afraid of breaking new technical developments rather than using them properly.						
What happens when I deal with new technical developments is ultimately under my control.						
I quickly take a liking to new technical developments.						
Whether I am successful in using modern technology depends largely on me.						

(1=does not apply at all; 6=fully applies)

Professional background

Professional group:

Health care and nursing ☐
Health care and pediatric nursing ☐
Nursing assistance ☐
Geriatric nursing ☐
Geriatric nursing assistance ☐
Academic nursing degree ☐
Education ☐
Other: ______________________________________

Do you have advanced specialty training?

Yes ☐
No ☐

Department (in which you primarily work):

Internal medicine ☐
Geriatrics ☐
Cardiology ☐
Nephrology ☐
Hematology and internal oncology ☐
Endocrinology ☐
Gastroenterology ☐
Pneumology ☐
Rheumatology ☐
Pediatrics ☐
Pediatric Cardiology ☐
Neonatology ☐
Pediatric surgery ☐
Pulmonary and bronchial medicine ☐
General surgery ☐
Trauma surgery ☐
Neurosurgery ☐
Vascular surgery ☐
Plastic surgery ☐
Thoracic surgery ☐
Cardiac surgery ☐
Urology ☐
Orthopedics ☐
Gynecology and obstetrics ☐
Obstetrics ☐
Ear, nose and throat medicine ☐
Ophthalmology ☐

Neurology ☐
General psychiatry ☐
Child and adolescent psychiatry ☐
Psychosomatics/Psychotherapy ☐
Nuclear medicine ☐
Radiation medicine ☐
Dermatology ☐
Dentistry, oral and maxillofacial surgery ☐
Intensive care medicine ☐
Other department: ______________________________________

What is the scope of your job (in percent)?

____

Work experience (in years, since beginning training):

____

Work experience at current job (in years):

____

Do you have personnel responsibility?

Yes ☐
No ☐

General job satisfaction – How would you rate the following statements?

	1	2	3	4	5	6
I have real joy in my work.						
I don't enjoy my work very much, but you shouldn't expect too much.						
My work is always in the same rut; there's nothing you can do about that.						
Would you say that your work is really interesting and satisfying?						
Does your job give you enough opportunities to use your skills?						
Are you satisfied with your opportunities for advancement?						
Are you satisfied with the pace of work?						
If you had to choose again, would you choose the same profession again?						

(1=does not apply at all; 6=fully applies)

Questions about your facility

Sponsorship of your hospital:

Public ☐
Non-profit/denominational ☐
Private ☐
Do not know exactly ☐

Number of beds:

Less than 100 ☐
Less than 200 ☐
Less than 300 ☐
Less than 400 ☐
Less than 500 ☐
Less than 600 ☐
Less than 700 ☐
Less than 800 ☐
800 or more ☐
Do not know exactly ☐

When you think about digitization at your own institution, how high do you rate the level of digitization overall and in your specialist department?

	1	2	3	4	5	6	7
Entire facility							
Own department							

(1=very low; 6=very high)

Gender of your leading person (meaning the direct leader with whom there is the most contact):

Male ☐
Female ☐
Diverse ☐

Age of your leading person (in years, estimate if necessary):

____

Importance of various job characteristics

How important do you personally consider the following characteristics in your everyday professional life?

	1	2	3	4	5	6
Interesting activities						
expansion of professional competencies and opportunities						
more time for individual patients						
completing tasks more quickly						

(1=not important at all; 6=very important)

Conclusion of the survey

If you have any final requests or comments, please feel free to share them:
